# Serum Starvation Accelerates Intracellular Metabolism in Endothelial Cells

**DOI:** 10.3390/ijms24021189

**Published:** 2023-01-07

**Authors:** Mario Lorenz, Raphaela Fritsche-Guenther, Cornelia Bartsch, Angelika Vietzke, Alina Eisenberger, Karl Stangl, Verena Stangl, Jennifer A. Kirwan

**Affiliations:** 1 Charité–Universitätsmedizin Berlin, Corporate Member of Freie Universität Berlin and Humboldt-Universität zu Berlin, Medizinische Klinik für Kardiologie und Angiologie, Campus Mitte, 10117 Berlin, Germany; 2 DZHK (German Centre for Cardiovascular Research), Partner Site Berlin, 10785 Berlin, Germany; 3 Metabolomics Platform, Berlin Institute of Health at Charité—Universitätsmedizin Berlin, 10117 Berlin, Germany

**Keywords:** metabolism, serum starvation, endothelial cells, HUVEC, flux analysis, labeling

## Abstract

Periods of low energy supply are challenging conditions for organisms and cells during fasting or famine. Although changes in nutrient levels in the blood are first sensed by endothelial cells, studies on their metabolic adaptations to diminished energy supply are lacking. We analyzed the dynamic metabolic activity of human umbilical vein endothelial cells (HUVECs) in basal conditions and after serum starvation. Metabolites of glycolysis, the tricarboxylic acid (TCA) cycle, and the glycerol pathway showed lower levels after serum starvation, whereas amino acids had increased levels. A metabolic flux analysis with ^13^C-glucose or ^13^C-glutamine labeling for different time points reached a plateau phase of incorporation after 30 h for ^13^C-glucose and after 8 h for ^13^C-glutamine under both experimental conditions. Notably, we observed a faster label incorporation for both ^13^C-glucose and ^13^C-glutamine after serum starvation. In the linear range of label incorporation after 3 h, we found a significantly faster incorporation of central carbon metabolites after serum starvation compared to the basal state. These findings may indicate that endothelial cells develop increased metabolic activity to cope with energy deficiency. Physiologically, it can be a prerequisite for endothelial cells to form new blood vessels under unfavorable conditions during the process of angiogenesis in vivo.

## 1. Introduction

Metabolic adaptations are essential to cope with a diminished supply of nutrients and energy. In humans, fasting induces major changes in the blood metabolome [1,2]. On the cellular level, metabolism is highly flexible and is being continuously adjusted in response to changing physiological environments. The vascular wall of blood vessels consists of a single layer of endothelial cells. These cells are primarily affected by changes in blood-derived nutrients. However, metabolic studies in human endothelial cells are sparse. In particular, studies of metabolic adaptations to conditions of limited nutrient supply are still missing. Human umbilical vein endothelial cells (HUVECs) are a widely used cell model to study molecular mechanisms of endothelial function, including responses to (patho)physiological stimuli [3].

Here, we measured the dynamic metabolic activity of HUVECs using a pulsed stable isotope-resolved metabolic analysis (pSIRM) with the application of labeled ^13^C-carbons. The time kinetics for the uptake and turnover of essential cellular nutrients were assessed under basal conditions and after serum starvation. Endothelial cells are characterized by their high rate of glycolysis, allowing for rapid adenosine triphosphate (ATP) production and the generation of precursors for biomass synthesis [4]. In addition, glutamine, as an energy source, is essential for cellular proliferation [5,6]. Using ^13^C-glucose or ^13^C-glutamine, we traced the incorporation of the labeled carbons into glycolysis or tricarboxylic acid (TCA) cycle intermediates, respectively, and analyzed the effects of serum starvation as a model for reduced cellular energy supply. Notably, we found that, compared to basal conditions, serum starvation accelerates rather than decreases the time kinetics of intracellular metabolite turnover.

## 2. Results

**Figure 1:**
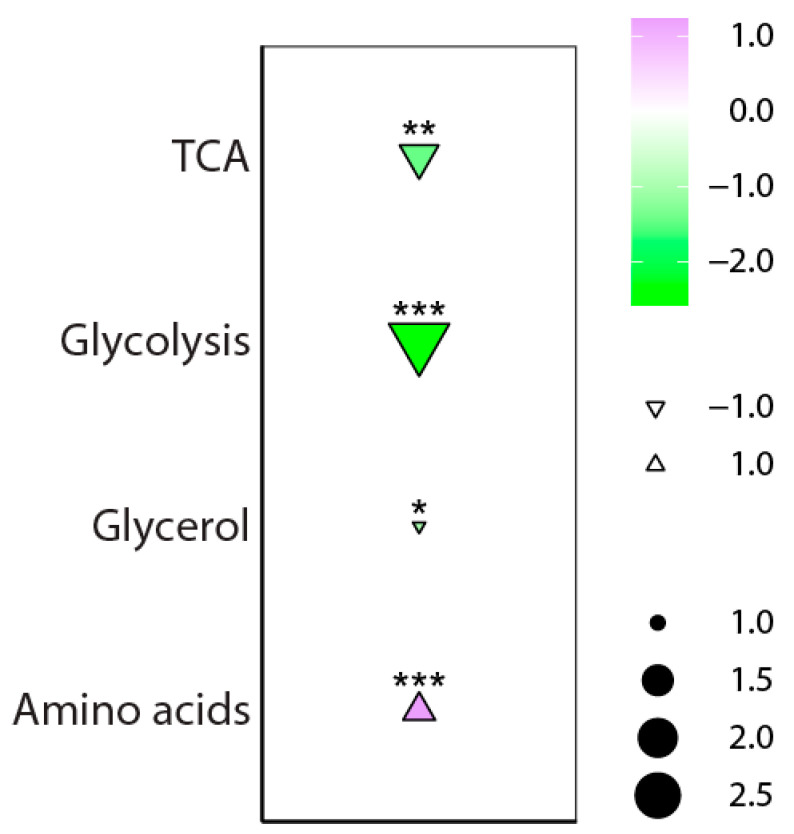
Levels of detected central carbon metabolites in HUVEC cells after serum starvation for 24 h compared to basal conditions. Metabolites were minimum/maximum-normalized and grouped according to the biological classes to show the overall effects of serum starvation. The colors and triangles indicate the direction and effect size (serum starvation relative to basal). The sizes of the dots indicate log2 fold changes in the means of the normalized peak areas of serum starvation versus the basal condition. Significance was calculated using the Mann–Whitney U test. Significant values: *p* < 0.05 (*), *p* < 0.01 (**) and *p* < 0.001 (***). *n* = 9 basal and *n* = 10 serum-starved. TCA: tricarboxylic acid cycle.

For our metabolomics approach, all samples were run in scan mode to obtain both targeted and untargeted metabolic profiles. Cells were serum-starved or not for 24 h before labeling. In a first steady-state approach, we selected 27 key central carbon metabolites that we targeted in previous studies [7,8,9]. These metabolites (4 glycolysis metabolites, 5 TCA metabolites, 3 metabolites from the glycerol pathway, and 15 amino acids) were detected in HUVECs under basal conditions as well as after serum starvation (Supplementary Table S1). Amino acids had significantly higher levels in serum-starved cells compared to the basal conditions (*p* < 0.05). In contrast, the metabolites of the TCA cycle (*p* < 0.001), glycolysis (*p* < 0.001), and the glycerol pathway (*p* < 0.05) showed significantly lower levels after serum starvation (Figure 1; Supplementary Figure S1).

Both increased or reduced cellular metabolic activity (the sum of metabolite production or accumulation) can result in altered metabolite levels after serum starvation. We therefore performed a metabolic flux analysis with ^13^C-glucose and ^13^C-glutamine labeling to trace metabolic compounds over time. In initial experiments, we identified appropriate time points for label incorporation under both experimental conditions (Supplementary Figures S2–S4). Early time points (up to 20 min for ^13^C-glucose and up to 60 min for ^13^C-glutamine) resulted in rather low incorporation rates (Supplementary Figure S2). Longer periods of up to 45 min for ^13^C-glucose and up to 180 min for ^13^C-glutamine increased label incorporation for glycolysis and TCA cycle metabolites. However, incorporation was still in the linear range for most of the metabolites (Supplementary Figure S3). Extending the labeling times to 30 h for ^13^C-glucose and to 8 h for ^13^C-glutamine resulted in a plateau phase for pyruvic acid and lactic acid (^13^C-glucose labeling) as well as for citric acid, succinic acid, fumaric acid, and malic acid (^13^C-glutamine labeling under basal conditions) (Supplementary Figure S4). The most striking finding, however, was a faster label incorporation for both ^13^C-glucose and ^13^C-glutamine after serum starvation throughout all time points (Supplementary Figures S2–S4).

**Figure 2:**
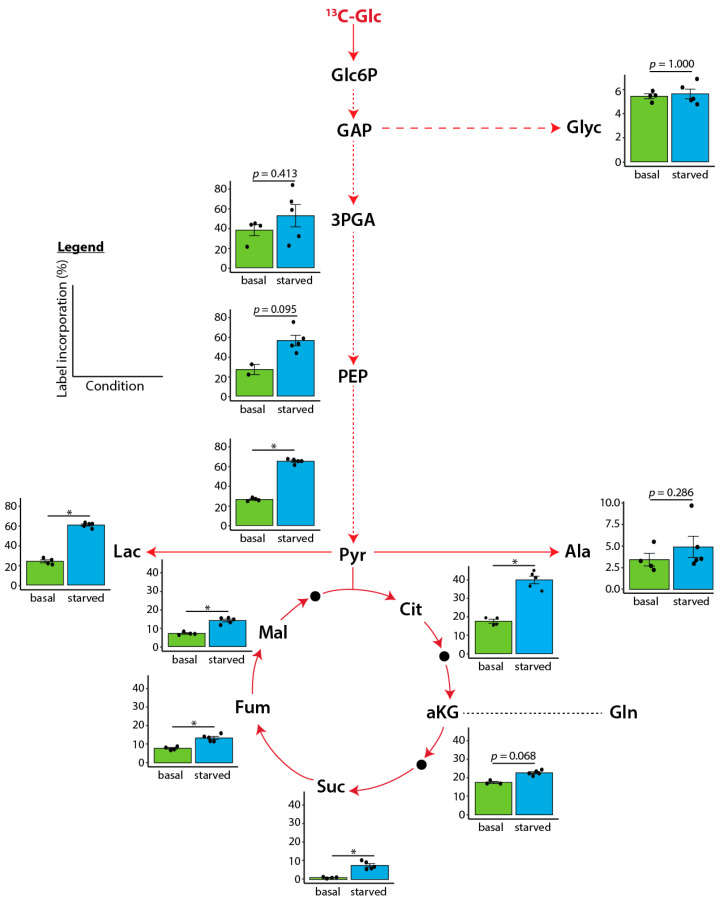
Incorporation of central carbon metabolites after labeling with ^13^C-glucose (Glc) for 3 h. Bar charts show the individual values and the means ± SEM of the label incorporation (in %). Significance was calculated using the Mann–Whitney U test. Significance levels are * *p* < 0.05 or as indicated. HUVECs from *n* = 4 different donors for basal conditions (except of *n* = 2 for PEP) and *n* = 5 for serum starvation. Abbreviations (from top to bottom): Glc: Glucose. Glc6P: glucose-6-phosphate. GAP: glyceraldehyd-3-phosphate. Glyc: glycerol. 3PGA: glyceric-acid-3-phosphate. PEP: phosphoenolpyruvic acid. Pyr: pyruvic acid. Lac: lactic acid. Ala: alanine. Cit: citric acid. Mal: malic acid. Fum: fumaric acid. aKG: alpha-ketoglutaric acid. Gln: glutamine. Suc: succinic acid.

To assess the results in a broader context, we measured label incorporation in the linear range after 3 h with HUVECs from *n* = 4–5 different donors. A faster incorporation of ^13^C-labeled glucose was observed into the glycolytic compounds glyceric-acid-3-phosphate, phosphoenolpyruvic acid, pyruvic acid, and lactic acid after serum starvation. In addition, the TCA metabolites citric acid, alpha-ketoglutaric acid, succinic acid, fumaric acid, and malic acid showed higher ^13^C-glucose incorporation. The amino acid alanine showed a slightly faster ^13^C-labeled incorporation after serum starvation, whereas glycerol was not affected (Figure 2).

**Figure 3:**
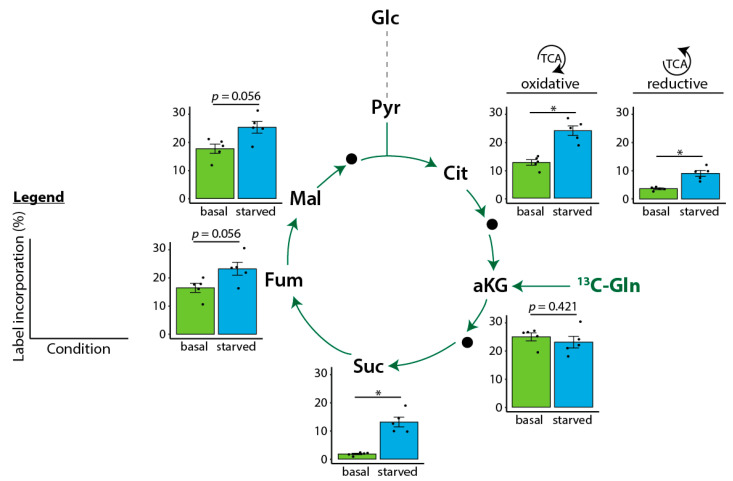
Incorporation of central carbon metabolites after labeling with ^13^C-glutamine (Gln) for 3 h. Bar charts present the individual values and the means ± SEM of the label incorporation (in %). Significance was calculated using the Mann–Whitney U test. Significance levels are * *p* < 0.05 or as indicated. HUVECs from *n* = 5 different donors. Abbreviations (from top to bottom): Glc: glucose. Pyr: pyruvic acid. Cit: citric acid. Mal: malic acid. Fum: fumaric acid. aKG: alpha-ketoglutaric acid. Gln: glutamine. Suc: succinic acid.

Glutamine is utilized as cellular energy source by conversion from glutamic acid to alpha ketoglutaric acid [10]. Serum starvation resulted in a faster label incorporation of ^13^C-labeled glutamine after 3 h into all TCA cycle metabolites, with the exception of alpha-ketoglutaric acid (Figure 3). While citric acid and succinic acid had higher label incorporation, fumaric acid and malic acid showed only a trend for faster label incorporation after serum starvation. Glutamine is also involved in lipid synthesis via reductive carboxylation [10]. Consistent with the results of the oxidative TCA cycle (Figure 2), the reductive TCA cycle, represented by reductive citric acid, showed faster label incorporation after serum starvation (Figure 3). An overview of the individual label incorporations in all HUVECs for both time points is provided in Supplementary Tables S2 and S3.

## 3. Discussion

Rapid changes in cellular metabolism are crucial for adaptations to altered physiological conditions. Our study revealed a faster incorporation of ^13^C-glucose and ^13^C-glutamine into central carbon metabolites in primary endothelial cells after serum starvation. This is a rather unexpected finding, since it could be expected that cells would rather reduce their metabolic activities with lower amounts of available nutrients.

Although HUVECs are a widely used cell model to study cardiovascular molecular mechanisms, metabolic labeling experiments are limited in these cells. The present studies used only one time point for labeling with a single nutrient. After labeling with ^13^C-glucose, laminar shear stress stimulated the biosynthesis of a structural component of the endothelial glycocalyx in HUVECs through altered intracellular glucose metabolism [11]. The growth-factor-induced angiogenic activation of HUVECs stimulated a high flux of glucose through the pentose phosphate pathway (PPP) as well as increased glycogen deposits and a high glycogen turnover after labeling with ^13^C-glucose [12]. Whereas proliferation was glutamine-dependent, cellular migration relied on glucose after labeling with ^13^C-glucose and ^13^C-glutamine. Glutamine was required in HUVECs, mainly as a nitrogen source for the generation of biomass [13]. However, after forming a complex vascular network on a Matrigel, glycolysis decreased and fatty acid oxidation increased, as shown through ^13^C-palmitic acid and ^13^C-glucose labeling [14]. In addition, serine synthesis proved to be essential to maintain mitochondrial respiration and homeostasis after the labeling of HUVECs with ^13^C-serine and ^13^C-glucose [15]. However, no metabolic flux analysis over time was performed in any of these studies. Notably, metabolic incorporation studies under conditions of cellular starvation are lacking.

The vascular system consists of a network of blood vessels lined by endothelial cells that supply the surrounding tissues with nutrients and oxygen. In the case of deficiency, endothelial cells are required to form new blood vessels (angiogenesis) in a short time. This process involves rapid changes in metabolic activities to cope with altered physiological demands [16]. During angiogenesis and repair processes, endothelial cells have to increase their metabolic activity for the biosynthesis of proteins, lipids, and nucleotides to enable enhanced proliferation and migration [17]. Due to their manifold physiological functions in vivo, the metabolism of endothelial cells is highly flexible [18]. Since glycolysis is associated with a faster rate of cellular ATP production (as opposed to mitochondrial respiration), endothelial cells are known to be highly glycolytic [16]. This distinctive feature also persists in cell culture [19]. In addition, endothelial cells rely on glutamine as a cellular energy source [13]. We therefore chose ^13^C-glucose and ^13^C-glutamine as nutrients for our labeling experiments. Serum starvation impairs the metabolic activity of the cell and therefore served as a model to study the biological impact of a diminished nutrient supply.

In our study, the levels of metabolites from glycolysis, the oxidative TCA cycle, and the glycerol pathway were reduced in starved cells compared to basal conditions, whereas the levels of amino acids were increased. During starvation, amino acid levels are regulated by the general amino acid control (GAAC) pathway [20]. In response to declining levels, activation transcription factor 4 (ATF4) upregulates amino acid biosynthesis and controls amino acid transporter genes [21]. Angiogenesis is promoted by the YAP/TAZ-TEAD transcriptional module in endothelial cells, leading to the upregulation of amino acid transporters and the activation of mTORC1 signaling [22]. This could explain why we observed increased amino acid pools after serum starvation. Primary cells cease proliferation after extended periods of starvation. Our observation of diminished glycolysis after serum starvation is in line with another study that revealed lower glycolysis in nonproliferating endothelial cells [14].

Changes in intracellular metabolite levels could originate from either increased or decreased metabolic activity in the cell (the net effect of metabolite production or accumulation and consumption). Since steady-state approaches do not provide insights into metabolic changes over time, we therefore performed, for the first time, a metabolic flux analysis at different time points in primary endothelial cells. Under basal conditions, both glucose and glutamine were utilized in endothelial cells. This indicates high energy and biomass demand for cellular proliferation [20]. In addition, we observed a faster incorporation plateau for ^13^C-glutamine compared to ^13^C-glucose, both under basal conditions and after serum starvation, reflecting the higher significance of glutamine for cellular biomass production in endothelial cellsThe main result of our study was, however, the faster label incorporation of both ^13^C-glucose and ^13^C-glutamine after serum starvation at all time points, which was also verified using HUVECs from five different donors in the linear phase of label incorporation. This finding points to a higher metabolic activity of endothelial cells after serum starvation compared to the basal condition.

There are a number of potential explanations for this result. Serum starvation represents a serious challenge to the cell that can eventually lead to apoptosis. In order to survive, cells would make an effort to produce metabolic intermediates to cope with a diminished energy supply. In vivo, endothelial cells have to form new blood vessels during periods of diminished nutrient supply and hypoxia to supply the surrounding tissues with nutrients and oxygen [23]. To meet this requirement, they have to remain viable and metabolically active, even under unfavorable conditions. A reduction in nutrients (as mimicked by serum starvation in the cell culture) may therefore be the trigger to increase, rather than decrease, the metabolic activity of endothelial cells. This may only last for a limited amount of time before cellular reserves are depleted and angiogenesis is terminated in vivo. During starvation, cells use gluconeogenesis to provide energy to prolong cellular viability [24]. The main substrates for this process are amino acids. Our results revealed an increased pool of amino acids and an enhanced label incorporation into alanine after serum starvation. Since angiogenesis involves both the proliferation and migration of endothelial cells, glutamine as well as glucose are needed as energy sources. In support of the hypothesis to maintain cellular viability, the reductive TCA cycle via citric acid showed a significantly faster label incorporation after serum starvation in our study, suggesting a rapid synthesis of fatty acids, which are important for membrane stability.

In summary, we found a faster metabolic incorporation of cellular nutrients after serum starvation compared to the basal state. This applied to both glycolysis and TCA cycle metabolites, as measured by ^13^C-label incorporation, and all investigated time points. The rationale for this unexpected finding could reflect the need for endothelial cells to form new blood vessels in poor physiological conditions in vivo.

## 4. Materials and Methods

### 4.1. Isolation and Cultivation of HUVECs

HUVECs were isolated as previously described [9]. Cells were cultured in M199 (Thermo Fisher Scientific, Waltham, MA, USA) supplemented with 20% FCS (fetal calf serum, Biochrom, Merck, Berlin, Germany), 12 µg/mL ECGS (endothelial cell growth supplement, PromoCell, Heidelberg, Germany), 1 U/mL heparin (Biochrom, Merck, Berlin, Germany), 2 mM L-glutamine (Thermo Fisher Scientific, Waltham, MA, USA), 5 µg/mL ascorbic acid (Sigma Aldrich, St. Louis, MO, USA), 5 µg/mL glutathione ((Sigma Aldrich, St. Louis, MO, USA), 100 U/mL penicillin (Thermo Fisher Scientific, Waltham, MA, USA), 100 µg/mL streptomycin (Thermo Fisher Scientific, Waltham, MA, USA), and 50 ng/mL amphotericin B (Biochrom, Merck, Berlin, Germany). The medium contained 5.5 mM glucose and 2.68 mM glutamine. Cells were incubated in a humidified atmosphere with 5 % CO_2_ in the air at 37 °C. The isolation of HUVECs conformed to local university guidelines and with the principles outlined in the Declaration of Helsinki. The isolation of HUVECs was approved by the Charité University Hospital Ethics Committee (EA2/017/13), and the mothers provided their written informed consent.

### 4.2. ^13^C Labeling and Extraction

First, 10^6^ cells were seeded in 10 cm^2^ culture dishes and grown until confluence. Then, 24 h before the experiments, cells were washed twice with phosphate buffered saline (PBS, Thermo Fisher Scientific, Waltham, MA, USA) and incubated in M199 with either 20% or 0.5% FCS. At the beginning of the experiment (time zero), cells were changed to the label medium (M199 without glucose and glutamine, Caisson Laboratories, #MDP01–10X1L, Smithfield, UT, USA). The label medium was supplemented with 5.5 mM glucose for labeling with ^13^C-glutamine, 2.68 mM glutamine for labeling with ^13^C-glucose, or 5.5 mM glucose and 2.68 mM glutamine for no labeling. Labeling was performed with 5.5 mM ^13^C-glucose (U13C6 D-glucose, Cambridge Isotope Laboratories, Tewksbury, MA, USA) or with 2.68 mM ^13^C-glutamine (^13^C L-glutamine, Cambridge Isotope Laboratories, Tewksbury, MA, USA). Cells were incubated for the indicated time points with the corresponding label media. At the ends of the experiments, cells were rapidly washed (20 s) with wash buffer (140 mM NaCl and 5 mM HEPES, pH 7.4, room temperature) containing either ^13^C-glucose/^12^C-glutamine, ^12^C-glucose/^13^C-glutamine, or ^12^C-glucose/^12^C-glutamine (zero time point) at the same concentration as in the original media. Cells were lysed with 5 mL of an ice-cold 50% methanol (MeOH, Th. Geyer, Berlin, Germany) solution containing 2 µg/mL cinnamic acid for use as an internal standard (Sigma Aldrich, St. Louis, MO, USA). Immediately after the MeOH solution was added to the culture plate, lysates were scraped into the methanol solution, and the methanolic lysates were collected and stored at −20 °C. Subsequently, 4 mL of chloroform (CHCl_3_, VWR International, Radnor, PA, USA), 1.5 mL of MeOH, and 1.5 mL of water (H_2_O, VWR International, Radnor, PA, USA) were added to the methanolic cell extracts, shaken for 60 min at 4 °C, and centrifuged at 4149× *g* for 10 min to separate the phases. The polar phase (6 mL) was collected and dried at 30 °C at a speed of 1550× *g* at 0.1 mbar using a rotational vacuum concentrator (RVC 2–33 CDplus, Christ, Osterode am Harz, Germany). Samples were pooled after extraction and used as a quality control sample to measure the technical variability of the instrument. They were prepared alongside the samples in the same way. After drying, samples were split by adding 600 µL of 20% MeOH to the dried extracts and shaking for 60 min at 4 °C, followed by centrifugation at maximum speed (18,213× *g*) for 10 min. Two 280 µL aliquots per sample were then dried under vacuum; one was analyzed and the other kept as a backup. For the determination of the appropriate labeling times, *n* = 3 technical replicates (pools of HUVECs from different donors) were used. Five individual HUVECs from different donors were used in the main experiment.

### 4.3. GC-MS Metabolomics Measurement of Key Central Carbon Pathway Metabolites

All polar cell extracts were stored dry at −80 °C until analysis. Extracts were removed from the freezer and dried in a rotational vacuum concentrator for 60 min before further processing to ensure there was no residual water, which may influence the derivatization efficiency. Dried extracts were dissolved in 15 µL of a methoxyamine hydrochloride (Sigma Aldrich, St. Louis, MO, USA) solution (40 mg/mL in pyridine, Sigma Aldrich, St. Louis, MO, USA) and incubated for 90 min at 30 °C with constant shaking, followed by the addition of 50 µL of N-methyl-N-[trimethylsilyl]trifluoroacetamide (MSTFA, Machery-Nagel, Düren, Germany) and incubation at 37 °C for 60 min. The extracts were centrifuged for 10 min at 10,000× *g*, and aliquots of 25 µL were transferred into glass vials for GC-MS measurement. An identification mixture for compound identification was prepared and derivatized in the same way, and an alkane mixture for reliable retention index calculation was included [25]. A metabolite analysis was performed on a Pegasus 4D GCxGC TOFMS-System (LECO Corporation, St. Joseph, MN, USA) complemented by an auto-sampler (Gerstel MPS DualHead with CAS4 injector, Mühlheim an der Ruhr, Germany). The samples were injected in split mode (split 1:5, injection volume: 1 µL) in a temperature-controlled injector with a baffled glass liner (Gerstel, Mühlheim an der Ruhr, Germany). The following temperature program was applied during sample injection: for 2 min, the column was allowed to equilibrate at 68 °C; then the temperature was increased by 5 °C/min until 120 °C; then it was increased by 7 °C/min up to 200 °C; then it was increased by 12 °C/min up to a maximum temperature of 320 °C, which was then held for 7.5 min. Gas chromatographic separation was performed on an Agilent 7890 (Agilent Technologies, Santa Clara, CA, USA) equipped with a VF-5ms column (Agilent Technologies, Santa Clara, CA, USA) with a 30 m length, a 250 µm inner diameter, and a 0.25 µm film thickness. Helium was used as the carrier gas, with a flow rate of 1.2 mL/min. The spectra were recorded in a mass range of 60 to 600 *m*/*z* with 10 spectra/s. The GC-MS chromatograms were processed with ChromaTOF software (LECO Corporation, St. Joseph, MN, USA), including a baseline assessment, peak picking, and a computation of the area. Targeted metabolites from the central carbon metabolism were selected for further data analysis (Supplementary Table S1). Label incorporation was determined according to the mass pairs after the application of ^13^C-glucose or ^13^C-glutamine (Supplementary Table S4) [26]. The data were exported and merged with an in-house-written R script. For the determination of the metabolite quantities (Figure 1) the 3 h time points from glucose and glutamine labeling were merged (*n* = 9 from the basal condition and *n* = 10 from the serum-starved condition; one replicate was lost in the basal condition due to a misinjection). The peak area of each metabolite was calculated by normalization to the sum of the area per sample. Individual derivatives were summed up. Significance was analyzed using the Mann–Whitney U test. The reason for combining the two datasets was that the pool sizes of the measured compounds should not change over time with regard to the added label media. When analyzing the split batches, median RSD values of 34% and 42% for glutamine and 23% and 25% for glucose for 20% FCS (basal) and 0.5% FCS (serum-starved) were found, respectively. The combined datasets led to median RSD values of 37% and 39% for 20% FCS (basal) and 0.5% FCS (serum-starved), respectively.

## Data Availability

The data used to support the findings of the study are available from the corresponding author upon request.

## Supplementary Materials

The supporting information can be downloaded at: https://www.mdpi.com/article/10.3390/ijms24021189/s1.
