# Supplementary material for: Serum Starvation Accelerates Intracellular Metabolism in Endothelial Cells
Source: Int J Mol Sci. 2023 Jan 7;24(2):1189. doi: 10.3390/ijms24021189 (PMC9863832; doi:10.3390/ijms24021189)
Supplement: Supplementary file 1 [file ijms-24-01189-s001.zip › ijms-2054323-supplementary.pdf]

International Journal of
Molecular Sciences

MDPI

Article

Serum starvation accelerates intracellular metabolism in endothelial cells

Mario Lorenz [1,2,†], Raphaela Fritsche-Guenther [3,†,*], Cornelia Bartsch [1], Angelika Vietzke [1], Alina Eisenberger [3], Karl Stangl [1], Verena Stangl [1,2,‡] and Jennifer A. Kirwan [3,‡]

1 Charité–Universitätsmedizin Berlin, corporate member of Freie Universität Berlin and Humboldt-Universität zu Berlin, Medizinische Klinik für Kardiologie und Angiologie, Campus Mitte, 10117 Berlin, Germany
2 DZHK (German Centre for Cardiovascular Research), partner site Berlin, 10785 Berlin, Germany
3 Metabolomics Platform, Berlin Institute of Health at Charité – Universitätsmedizin Berlin, 10117 Berlin, Germany
* Correspondence: raphaela.fritsche@bih-charite.de
† These authors contributed equally to this work.
‡ These authors contributed equally to this work.

Supplementary material contains the following information:

Supplemental Table S1. List of metabolites and their corresponding derivatives and the biological groups used for reference search. AA: Amino acids; TCA: Tricarboxylic acid cycle; TMS: Trimethylsilyl derivatives; MeOX: Methoxyamine hydrochloride.

Supplemental Table S2. Label incorporation (in %) for ^{13}C-glucose and ^{13}C-glutamine after 0 hours (background) under both experimental conditions in HUVECs from 5 different donors.

Supplemental Table S3. Label incorporation (in %) for ^{13}C-glucose and ^{13}C-glutamine after 3 hours under both experimental conditions in HUVECs from 5 different donors.

Supplemental Table S4. Mass pairs (mass fragment m/z) analyzed after ^{13}C-glucose or ^{13}C-glutamine labeling of HUVEC.

Supplemental Figure S1. Levels of detected central carbon metabolites. Shown are the log2 scaled calculated ratio of serum starved to basal cells. Significance was calculated using the Mann-Whitney-U test. Significant values $p < 0.05$ (*), $p < 0.01$ (**). TCA: tricarboxylic acid cycle.

Supplemental Figure S2. Incorporation of central carbon metabolites after labeling in the presence of ^{13}C-glucose (A) or ^{13}C-glutamine (B) for the indicated time points.

Supplemental Figure S3. Incorporation of central carbon metabolites after labeling in the presence of ^{13}C-glucose (A) or ^{13}C-glutamine (B) for the indicated time points.

Supplementary Figure S4. Incorporation of central carbon metabolites after labeling in the presence of ^{13}C-glucose (A) or ^{13}C-glutamine (B) for the indicated time points.

Supplemental Table S1. List of metabolites and their corresponding derivatives and the biological groups used for reference search. AA: Amino acids; TCA: Tricarboxylic acid cycle; TMS: Trimethylsilyl derivatives; MeOX: Methoxyamine hydrochloride.

Group	Metabolite	detected as
AA	Alanine	2TMS, 3TMS
AA	ß-Alanine	2TMS, 3TMS
AA	Asparagine	2TMS
AA	Aspartic acid	2TMS, 3TMS
AA	Glycine	2TMS, 3TMS
AA	Isoleucine	1TMS, 2TMS
AA	Leucine	1TMS, 2TMS
AA	Lysine	3TMS
AA	Methionine	1TMS, 2TMS
AA	Phenylalanine	1TMS, 2TMS
AA	Proline	1TMS, 2TMS
AA	Serine	2TMS, 3TMS, 4TMS
AA	Threonine	2TMS, 3TMS
AA	Tyrosine	3TMS
AA	Valine	1TMS, 2TMS
Glycerol	Dihydroxyacetone phosphate	1MeOX(3TMS)
Glycerol	Glycerol	3TMS
Glycerol	Glycerol-3-phosphate	4TMS
Glycolysis	Glyceric-acid-3-phosphate	4TMS
Glycolysis	Lactic acid	2TMS
Glycolysis	Phosphoenolpyruvic acid	3TMS
Glycolysis	Pyruvic acid	1MeOX(1TMS)
TCA	Citric acid	4TMS
TCA	Fumaric acid	2TMS
TCA	alpha-Ketoglutaric acid	1MeOX(2TMS)
TCA	Malic acid	3TMS
TCA	Succinic acid	2TMS

Supplemental Table S2. Label incorporation (in %) for ^{13}C-glucose and ^{13}C-glutamine after 0 h (background) under both experimental conditions in HUVECs from 5 different donors.

**^{13}C-glucose labeling**										
**HUVEC No.**	**1**	**2**	**3**	**4**	**5**	**1**	**2**	**3**	**4**	**5**
**Condition**	**basal**					**starved**				
Pyruvic acid	0.0	0.0	0.6	1.5	0.4	1.9	0.0	1.1	0.6	0.0
Glyceric-acid-3-phosphate	3.8	1.9	7.1	6.6	5.9	0.0	0.0	0.0	0.0	0.3
Lactic acid	0.6	0.6	0.6	0.6	0.6	0.7	0.6	0.7	0.9	0.6
Citric acid	0.2	0.3	0.5	0.1	0.0	0.0	0.1	0.0	0.8	0.4
Fumaric acid	0.0	0.0	0.2	0.3	0.1	0.0	0.0	0.0	0.1	0.0
Succinic acid	0.0	0.4	0.0	0.0	0.0	0.2	0.0	0.0	0.0	0.0
alpha-Ketoglutaric acid	4.5	7.3	9.7	6.4	6.4	5.5	18.5	22.1	14.8	13.5
Malic acid	0.0	0.0	0.4	0.0	0.5	0.0	0.3	0.0	0.4	0.0
Alanine	0.5	0.4	0.4	0.3	0.5	0.4	0.5	0.4	0.2	0.1
Glycerol	4.4	4.4	4.6	4.5	4.7	5.1	5.6	4.7	4.3	4.8
Phosphoenolpyruvic acid	0.0	0.0	0.0	2.1	7.4	17.3	10.4	5.2	9.1	0.0

**^{13}C-glutamine labeling**										
**HUVEC No.**	**1**	**2**	**3**	**4**	**5**	**1**	**2**	**3**	**4**	**5**
**Condition**	**basal**					**starved**				
Citric acid oxy	0.4	0.0	0.2	0.9	NA	0.2	0.6	0.5	0.583	NA
Citric acid red	0.3	0.1	0.1	0.5	NA	0.1	0.2	0.1	0.158	NA
Fumaric acid	0.0	0.1	0.1	0.3	NA	0.1	0.1	0.7	0.096	NA
Succinic acid	NA	NA	0.1	0.0	NA	0.2	0.0	0.1	0.000	NA
alpha-Ketoglutaric acid	1.5	0.8	NA	0.0	NA	1.7	1.2	2.3	4.351	NA
Malic acid	0.1	0.0	0.0	0.1	NA	0.0	0.0	0.0	0.349	NA

NA: not available due to misinjections.

Supplemental Table S3. Label incorporation (in %) for ^{13}C-glucose and ^{13}C-glutamine after 3 h under both experimental conditions in HUVECs from 5 different donors.

**^{13}C-glucose labeling**										
**HUVEC No.**	**1**	**2**	**3**	**4**	**5**	**1**	**2**	**3**	**4**	**5**
**Condition**			**basal**					**Starved**		
Pyruvic acid	28.4	24.7	26.9	25.5	NA	61.3	65.2	65.3	67.4	66.7
Glyceric-acid-3-phosphate	45.1	21.3	42.7	43.8	NA	22.4	32.1	67.3	84.1	58.8
Lactic acid	27.0	21.5	24.9	20.3	NA	58.8	55.9	62.1	60.9	60.7
Citric acid	19.7	16.2	19.8	15.7	NA	34.5	43.7	37.2	45.7	41.9
Fumaric acid	8.3	6.9	8.9	7.3	NA	11.7	14.2	11.6	16.0	13.6
Succinic acid	1.4	0.9	1.2	0.5	NA	6.0	9.3	6.9	10.4	5.6
alpha-Ketoglutaric acid	Out	18.5	16.8	16.7	NA	22.2	22.2	24.2	20.8	23.3
Malic acid	6.9	6.3	8.2	7.0	NA	11.7	14.8	13.2	15.6	15.4
Alanine	5.5	2.7	3.3	2.2	NA	4.9	9.7	3.5	3.4	3.0
Glycerol	5.9	4.9	5.5	5.5	NA	6.9	6.2	4.8	5.2	5.1
Phosphoenolpyruvic acid	32.3	NA	21.9	NA	NA	75.4	58.7	53.3	51.5	43.7

**^{13}C-glutamine labeling**										
**HUVEC No.**	**1**	**2**	**3**	**4**	**5**	**1**	**2**	**3**	**4**	**5**
**Condition**			**basal**					**Starved**		
Citric acid oxy	15.1	14.3	9.3	13.1	12.3	26.7	28.9	19.1	25.3	21.8
Citric acid red	4.3	4.1	2.7	3.6	3.5	9.9	12.2	6.3	9.8	7.5
Fumaric acid	17.9	20.1	10.6	17.7	16.1	23.6	30.6	16.3	23.8	21.9
Succinic acid	2.4	2.2	0.9	2.0	1.7	12.7	19.2	10.0	14.6	9.8
alpha-Ketoglutaric acid	26.4	27.1	19.5	25.2	26.7	21.0	30.4	18.0	24.1	22.1
Malic acid	20.1	21.0	11.8	18.6	16.6	26.7	31.1	18.3	25.1	24.7

NA: not available due to misinjections. Out: Significant outlier analyzed with Grubbs test (https://www.graphpad.com/quickcalcs/Grubbs1.cfm).

Supplemental Table S4. Mass pairs (mass fragment m/z) analyzed after ^{13}C-glucose or ^{13}C-glutamine labeling of HUVEC.

**Metabolite**	**Unlabeled**	**Labeling with ^{13}C-glucose**	**Labeling with ^{13}C-glutamine**
Glycerol	218	221	-
Alanine	188	190	-
Citric acid	273	275	277
Fumaric acid	245	247	249
Glyceric-acid-3-phosphate	357	359	-
alpha-Ketoglutaric acid	200	202	204
Lactic acid	117	119	-
Malic acid	233	235	236
Phosphoenolpyruvic acid	369	372	-
Pyruvic acid	174	177	-
Succinic acid	247	249	251

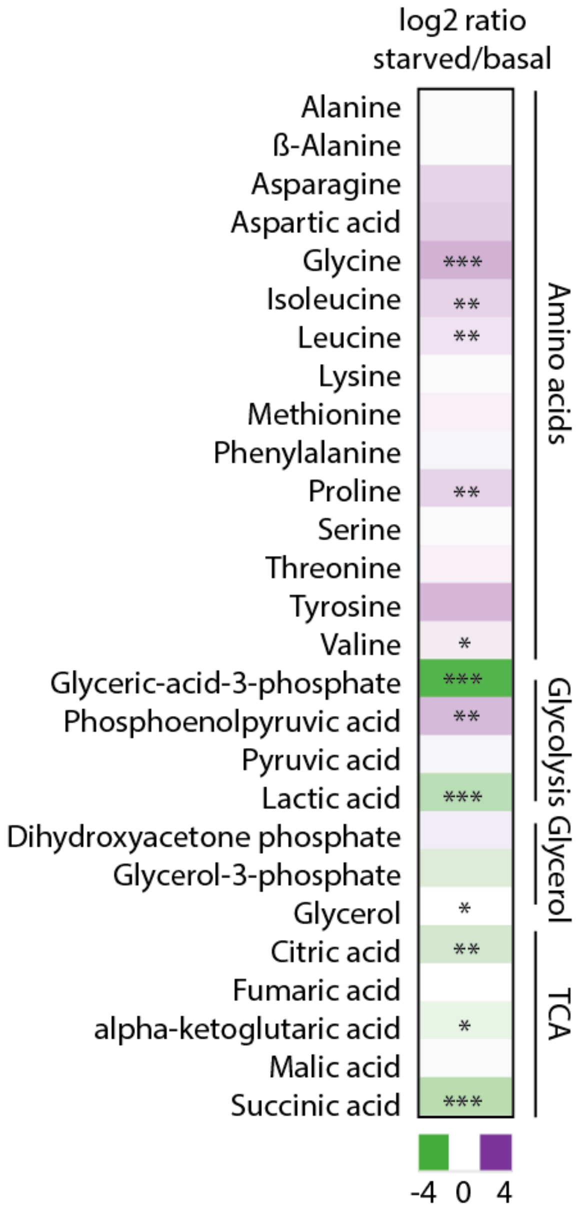

Supplemental Figure S1. Levels of detected central carbon metabolites. Shown are the log2 scaled calculated ratio of serum starved to basal cells. Cells were harvested after 3 h. Significance was calculated using the Mann-Whitney-U test. Significant values $p < 0.05$ (*), $p < 0.01$ (**) or $p<0.001$ (***). TCA: tricarboxylic acid cycle.

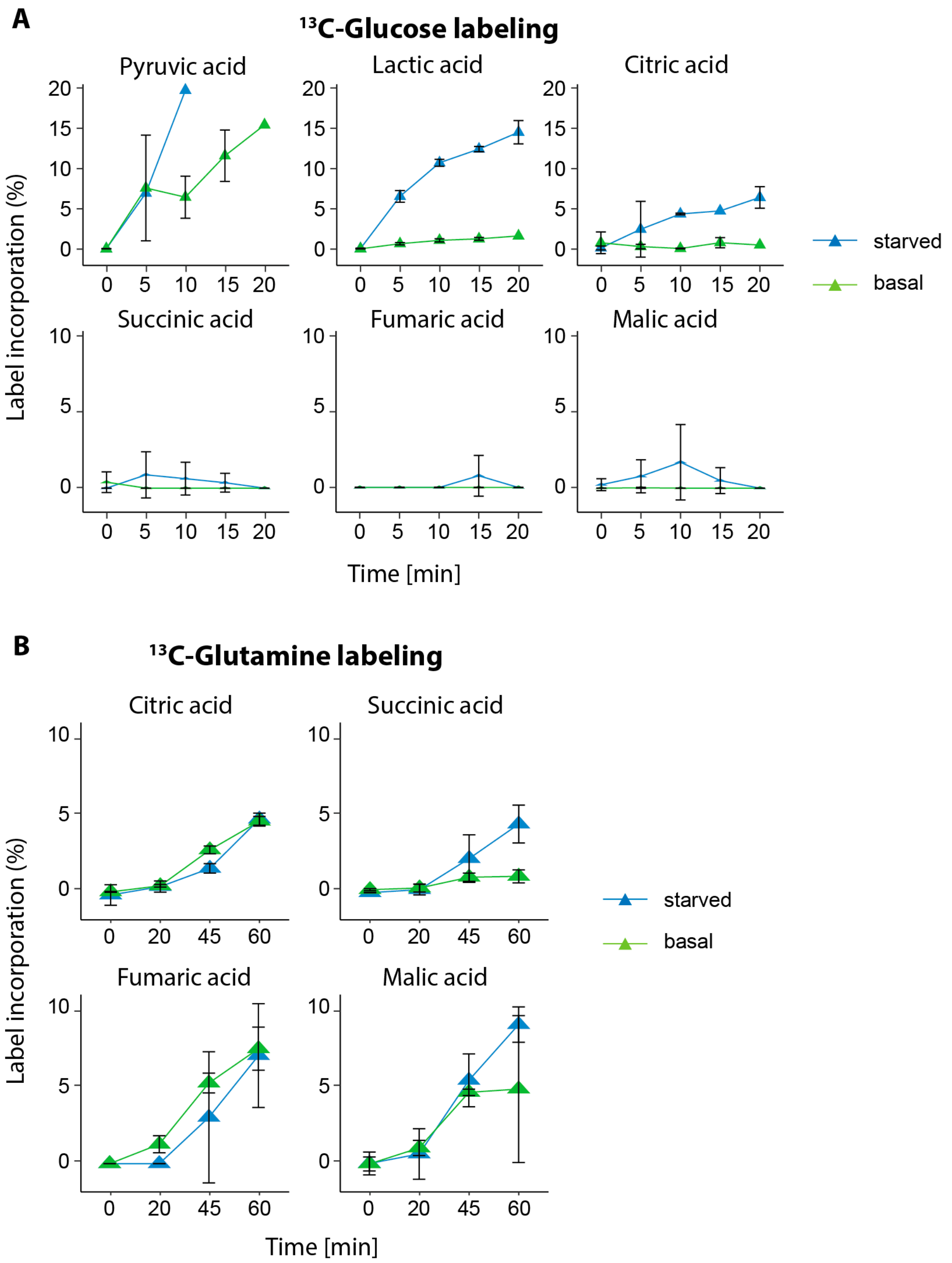

Supplemental Figure S2. Incorporation of central carbon metabolites after labeling in the presence of ^{13}C-glucose (A) or ^{13}C-glutamine (B) for the indicated time points.

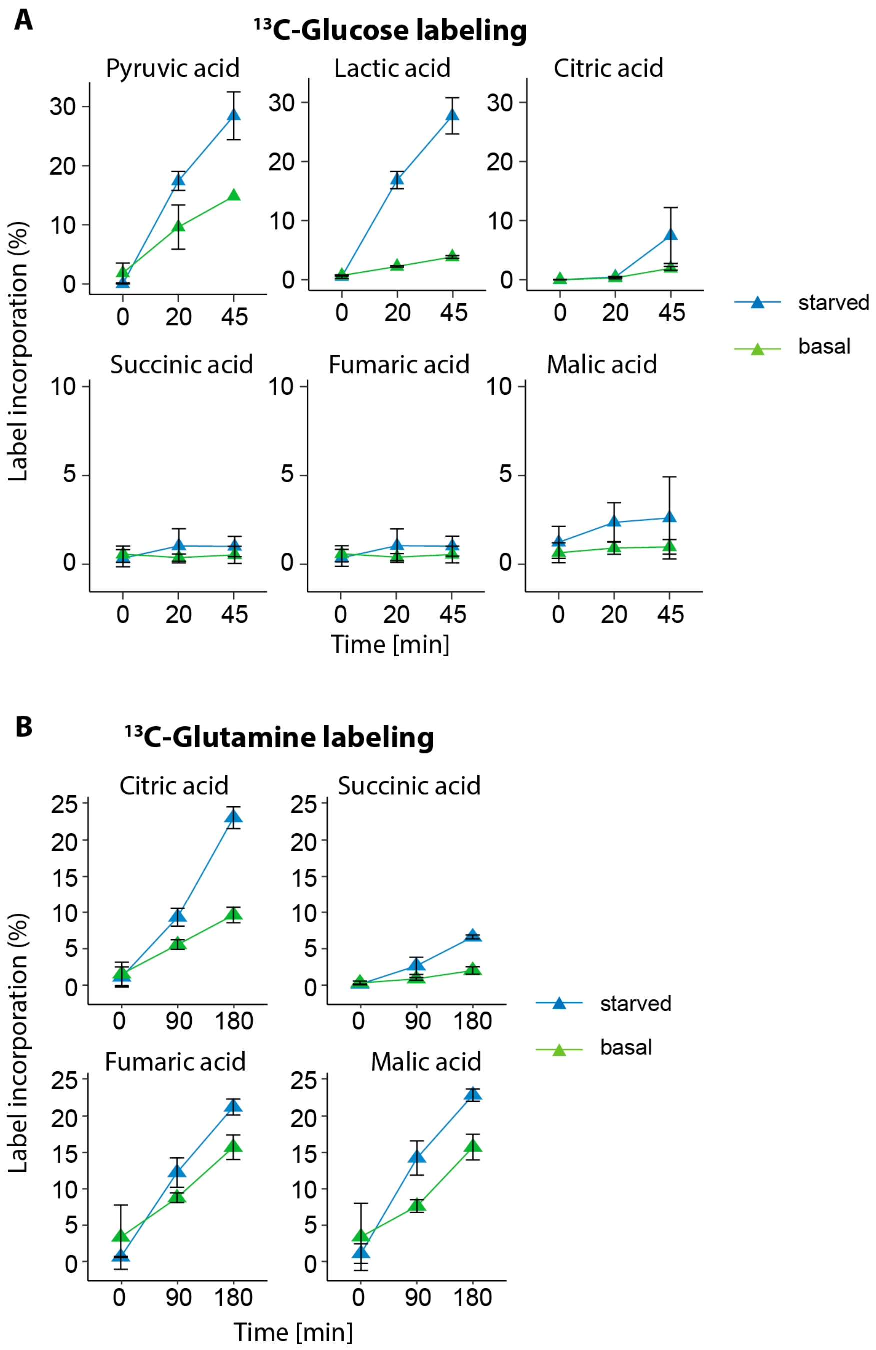

Supplemental Figure S3. Incorporation of central carbon metabolites after labeling in the presence of ^{13}C-glucose (A) or ^{13}C-glutamine (B) for the indicated time points.

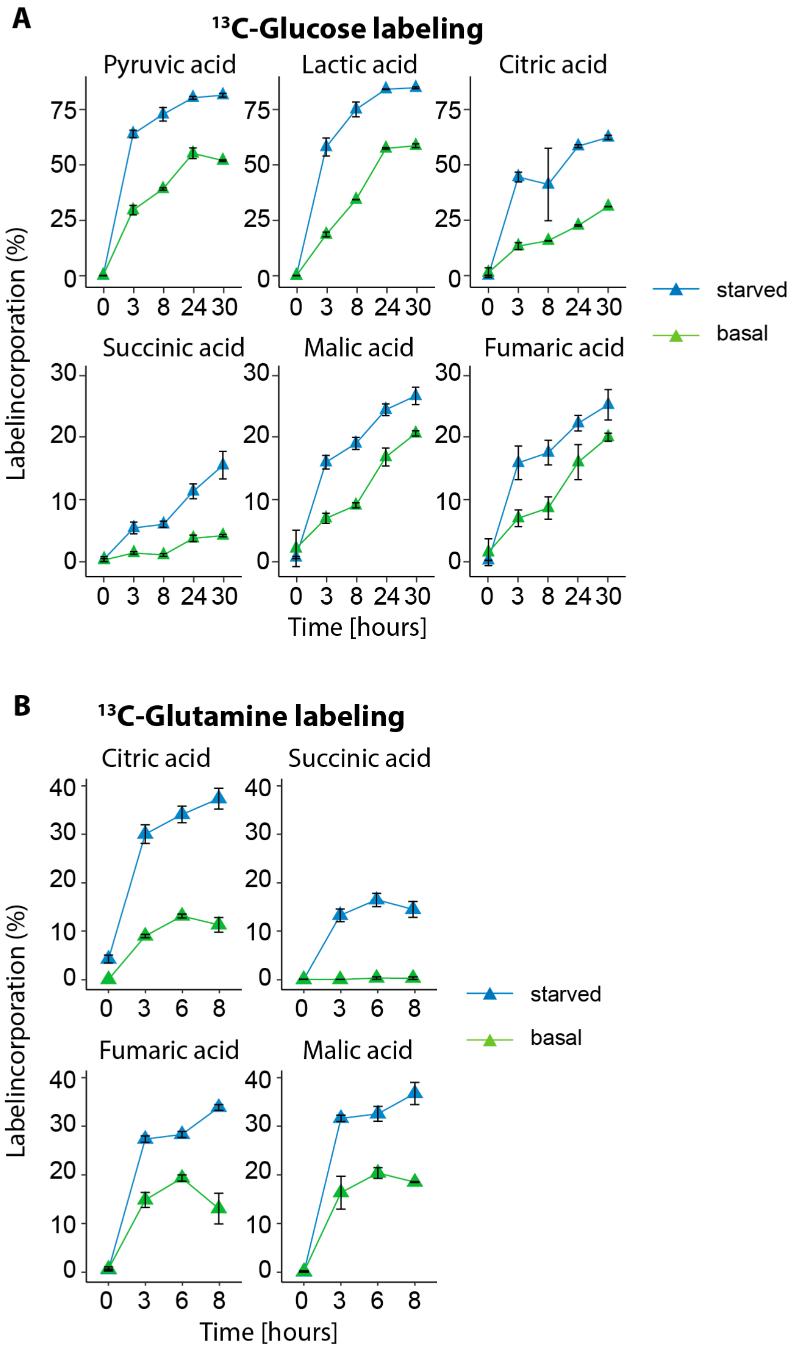

Supplementary Figure S4. Incorporation of central carbon metabolites after labeling in the presence of ^{13}C-glucose (A) or ^{13}C-glutamine (B) for the indicated time points.
